# Block-Diagonal Constrained Low-Rank and Sparse Graph for Discriminant Analysis of Image Data

**DOI:** 10.3390/s17071475

**Published:** 2017-06-22

**Authors:** Tan Guo, Xiaoheng Tan, Lei Zhang, Chaochen Xie, Lu Deng

**Affiliations:** College of Communication Engineering, Chongqing University, Chongqing 400044, China; xie_cc1@cqu.edu.cn (C.X.); ludeng@cqu.edu.cn (L.D.)

**Keywords:** dimensionality reduction, low-rank representation, sparse representation, block-diagonal, image classification

## Abstract

Recently, low-rank and sparse model-based dimensionality reduction (DR) methods have aroused lots of interest. In this paper, we propose an effective supervised DR technique named block-diagonal constrained low-rank and sparse-based embedding (BLSE). BLSE has two steps, i.e., block-diagonal constrained low-rank and sparse representation (BLSR) and block-diagonal constrained low-rank and sparse graph embedding (BLSGE). Firstly, the BLSR model is developed to reveal the intrinsic intra-class and inter-class adjacent relationships as well as the local neighborhood relations and global structure of data. Particularly, there are mainly three items considered in BLSR. First, a sparse constraint is required to discover the local data structure. Second, a low-rank criterion is incorporated to capture the global structure in data. Third, a block-diagonal regularization is imposed on the representation to promote discrimination between different classes. Based on BLSR, informative and discriminative intra-class and inter-class graphs are constructed. With the graphs, BLSGE seeks a low-dimensional embedding subspace by simultaneously minimizing the intra-class scatter and maximizing the inter-class scatter. Experiments on public benchmark face and object image datasets demonstrate the effectiveness of the proposed approach.

## 1. Introduction

With the rapid development of information technology, nowadays high precision sensors sense large-scale data, especially image data, all the time. These data often feature high dimensionality, and consist of redundant information or noise. How to analyze these high-dimensional data has attracted the interest of many researchers. Dimensionality reduction (DR) is a practical way to deal with this problem. DR aims to find a lower-dimensional embedding subspace where some desired properties can be preserved as much as possible [1,2,3]. The most well-known DR methods are, for example, principal component analysis (PCA) [4] and linear discriminate analysis (LDA) [5]. PCA is unsupervised, and applies orthogonal projection to maximize data variance. As a supervised method, LDA finds the linear projection axes on which the ratio between the between-class and within-class scatters is maximized. LDA cannot be directly applied to small size sample (SSS) problem because the within-class scatter matrix is singular. To avoid this problem, Li et al. [6] adopted the difference of between-class scatter and within-class scatter as the discriminating criterion for embedding learning. The method, termed maximum margin criterion (MMC), is simple and effective. However, these linear methods cannot reveal the essential structure of data with non-linear distributions. With kernel tricks, kernel principal component analysis (KPCA) [7] and kernel Fisher discriminate analysis (KFD) [8] were developed to handle data with non-linearity structures. Some manifold learning algorithms such as LLE [9], Isomap [10], and Laplacian eigenmaps (LE) [11] have also been presented to discover the intrinsic manifold structure in data.

Yan et al. [12] proposed a general DR framework called graph embedding (GE), where some DR methods, e.g., PCA, LDA, Isomap, LLE, and LE, could be considered as special instances under the framework. Based on GE, some discriminant DR methods, e.g., marginal Fisher analysis (MFA) [12], neighborhood preserving discriminate embedding (NPDE) [13] and sparse discriminate manifold embedding (SDME) [14], have been developed. The differences between these methods lie in the design of intrinsic and penalty graphs and the type of embedding. Graph construction has become the key of most GE-based DR methods. However, the way to establish high-quality graphs is an open problem. Common graph construction methods include *k*-nearest neighbor and *ε*-radius ball, both of which connect graph vertices with simple rules which are, however, highly sensitive to dataset noise and it is difficult to determine the parameters for real-world applications.

Sparse and low-rank models have been widely applied for visual analysis. Sparse representation (SR) and low rank representation (LRR) are utilized to construct the affinity matrix (or graph) [15,16,17,18,19]. They express each datum as a linear combination of all other data points, and use the representation coefficient to measure the similarity or neighborhood relationship of samples. Contrastively, sparse graphs are able to preserve local linear structure but lack global constraints. LRR takes the correlation structure of data into account, and finds a low-rank representation instead of a sparse one. The low-rank property has been proved to be effective in preserving global data structures.

The block-diagonal structure is often desired in affinity matrix construction. Ideally, the affinity matrix should be block-diagonal, and the inter-class affinities are all zeros [20]. However, SR and LRR can only generate a block-diagonal affinity matrix under restrictive conditions. For example, it has been shown that when the subspaces are independent, the solution to LRR is block-diagonal [20]. For a block-diagonal structured solution, Zhao et al. [21] incorporated least square regression and graph regularization to construct a sparse graph with block-wise constraint (SGB) for face representation. The method can well uncover the global structure of the multiple subsets of the data, and preserve the local intrinsic information. The performance can be further boosted by introducing the idea of ensemble learning [22]. Tang et al. [23] developed an extension of LRR termed as structure-constrained LRR (SC-LRR) for general disjoint subspace clustering by introducing an explicit structure constraint. Zhang et al. [24] presented a discriminative, structured low-rank method to explore structure information by incorporating an ideal-code regularization term. Li et al. [25] proposed to restrict the representation coefficients outside the diagonal block to be small to get a block-diagonal structure representation. Alternatively, Feng et al. [26] explicitly pursued a block-diagonal structure solution via a graph Laplacian constraint-based formulation. In [27], sparse graph-based discriminate analysis (SGDA) was developed by preserving the sparse connection in a block-structured affinity matrix with class-specific samples. Similarly, low-rank graph-based discriminate analysis (LGDA) [28] preserves the global structure in data using low-rank constraints. Meanwhile, low-rank and sparse graphs have been applied for semi-supervised learning [29,30]. A sparse and low-rank graph-based discriminate analysis (SLGDA) method was developed in [28] to purse block-diagonal structured affinity matrix with both sparsity and low-rank constraints. However, SGDA, LGDA and SLGDA consider intra-class affinity relationship class by class, which suffers from high computation costs. Besides, these methods find the representation of each sample using only the intra-class samples, which might not be able to reveal inter-class adjacent relationships. 

**Figure 1:**
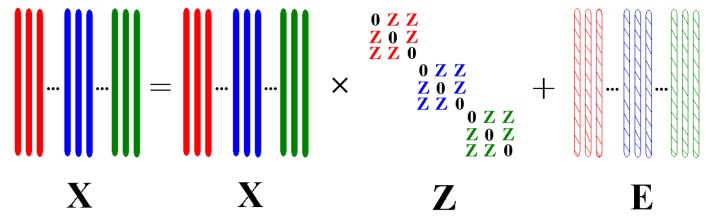
An example for desired block-diagonal constrained low-rank and sparse representation (BLSR). Samples in dataset X are encouraged to be represented by samples from the same class with noise E removed, and the representation matrix Z tends to have a block-diagonal structure.

In this paper, motivated by recent achievements in SR and LRR [15,16,17,18,19,20,21,22,23,24,25,26,27,28,29,30], we propose a novel supervised DR method, namely block-diagonal constrained low-rank and sparse-based embedding (BLSE). The model has two steps: first, a self-expressive model, i.e., block-diagonal constrained low-rank and sparse representation (BLSR) model is developed to reveal the intra-class and inter-class adjacent relationships among samples and discover the local and global structures latent in data. The desired representation learning example is shown in Figure 1. Here, different colors stand for samples of different classes. With the block-diagonal constraint, each sample is encouraged to be represented by intra-class samples. The obtained representation matrix Z tends to be block-diagonal and has good identification capability highlighting both intra-class similarities and inter-class differences. Due to these merits, the intra-class and inter-class representations obtained by BLSR are utilized to construct corresponding intra-class and inter-class graphs. 

**Figure 2:**
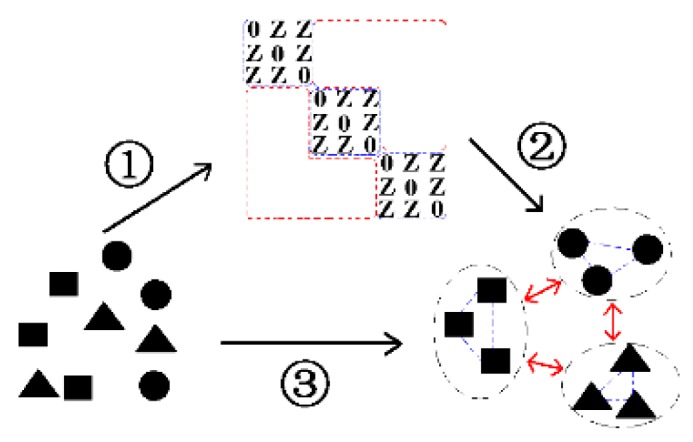
Illustration for block-diagonal constrained low-rank and sparse based embedding (BLSE) model. ① BLSR is applied to get the block-diagonal constrained low-rank and sparse representation of data. ② The representation results of BLSR is utilized to construct the intra-class and inter-class graphs. ③ BLSGE finds a low dimensional embedding with enhanced intra-class compactness and inter-class separation using the graphs.

Then, as shown in Figure 2, the block-diagonal constrained low-rank and sparse graph embedding (BLSGE) method finds a low-dimensional subspace with enhanced intra-class compactness and inter-class separation using the graphs. It is worth noting that there are some major differences between our BLSR model and the model presented in [23], although they have similar formulations. In that method, a weight matrix is defined to provide a moderate amount of correct information for the solution. Different from their strategy, we separately optimize the solution to be sparse, and develop an iteration method to explicitly optimize the diagonal elements of the solution to be large, and the rest ones to be small via a predefined block-diagonal mask matrix. Since our aim is to induce inter-class and inter-class graphs from the solution for further embedding learning, our strategy of promoting intra-class affinity weights large and inter-class affinity weights small is more applicable for the problem of interest. In sum, the main contributions of this paper are as follows:

(1) A self-expressive model, i.e., BLSR is devised by incorporating sparsity, low rankness as well as a novel block-diagonal constraint. BLSR can not only simultaneously capture the local and global structures, but also highlight both the intra-class similarities and inter-class differences of samples.
(2) With the intra-class and inter-class graphs derived from BLSR, BLSGE seeks an optimal feature space by simultaneously minimizing the intra-class scatter and maximizing the inter-class scatter. Generally, a novel supervised dimensionality reduction method namely BLSE is developed by taking the advantages of BLSR and GE framework.
(3) BLSE is applied for the dimensionality reduction and classification of visual data. Extensive experiments on the public face and object datasets verify the effective of proposed method.

The remainder of this paper is organized as follows: in Section 2, we briefly introduce some related works. In Section 3, we will introduce our BLSE model. Its two steps, i.e., BLSR and BLSGE, will be presented in detail. The experimental results are given in Section 4. Finally, we provide the discussion and conclusions in Section 5.

## 2. Related Works

Let us suppose a labeled dataset $X=\left[x_{1},x_{2},\ldots ,x_{n}\right]\in {ℜ}^{D\times n}$, D is the dimension of sample in original space. n is the number of training samples with $n_{k}\;\left(k=1,2,\ldots ,C\right)$ samples per class. The label of $x_{i}\left(i=1,2,\ldots ,n\right)$ is denoted as $l\left(x_{i}\right)$. Data points in X are ordered, as is common, in terms of their class labels. $Y=\left[y_{1},y_{2},\ldots ,y_{n}\right]\in {ℜ}^{d\times n}$ (typically d << D) is the lower dimensional projected data of X with projection matrix $V\in {ℜ}^{D\times d}$.

### 2.1. Low Rank and Sparse Representation

Low rank and sparse models have been seen a surge of interests in recent years, and been successfully exploited in many applications, such as subspace clustering [16,17], face recognition [25,31,32], head pose estimation [33], information processing [34,35,36,37], transfer learning [38,39], and extreme learning machine [40]. Low-rankness is an appropriate criterion to capture low-rank dimensional structure in high-dimensional data, and low-rank representation (LRR) is robust to sparse noise. Sparse representation (SR) has been shown good discrimination capacity. Low-rank and sparse representation is pursued to take the merits of both the two aspects. Learning the low-rank and sparse representation Z of dataset X on dictionary D can be formulated as follows:

(1) $$\underset{Z,E}{\min}\;rank\left(Z\right)+\lambda {\left\|E\right\|}_{l}+\beta {\left\|Z\right\|}_{0}\;s.t.\;X=DZ+E$$

where ${\left\|\cdot \right\|}_{l}$ is used to characterize noise E. It can be sparse noise ${\left\|E\right\|}_{0}$ or sample specific noise ${\left\|E\right\|}_{2,1}$. λ and β control the effects of noise term E and sparse representation term Z. Since the *l*_0_-norm and rank minimization problems are non-convex, the problem is NP-hard. Alternatively, rank function can be relaxed with nuclear norm, which is defined as the sum of the singular values of a matrix. The *l*_0_-norm can be surrogated with *l*_1_-norm. Thus, one can get the following relaxed optimization problem:

(2) $$\underset{Z,E}{\min}\;{\left\|Z\right\|}_{*}+\lambda {\left\|E\right\|}_{l}+\beta {\left\|Z\right\|}_{1}\;s.t.\;X=DZ+E$$

With dataset X itself as the dictionary, Reference [28] proposed the following optimization problem with sample-specific noise:

(3) $$\underset{Z,E}{\min}\;{\left\|Z\right\|}_{*}+\lambda {\left\|E\right\|}_{2,1}+\beta {\left\|Z\right\|}_{1}\;s.t.\;X=XZ+E,\mathrm{diag}\left(Z\right)=0$$

where diag (Z) represents the vector containing the diagonal elements of Z, and 0 is a zero vector. The obtained low rank and sparse representation matrix Z can be utilized to construct intrinsic graph for DR [28].

### 2.2. Graph Embedding

The GE framework provides a unified perspective to understand many DR algorithms [12]. In GE, an intrinsic graph G={X,W} that describes certain desired statistical or geometrical properties of data, and a penalty graph $G^{P}=\left\{X,W^{P}\right\}$ characterizes a statistical or geometric property which should be avoided need to be constructed. Both G and $G^{P}$ are undirected weighted graphs. X is the vertex set. $W\in {ℜ}^{n\times n}$ and $W^{p}\in {ℜ}^{n\times n}$ are the weight matrices. 

Assuming that the low-dimensional vector representations of the vertices can be obtained from a linear projection as $y=V^{T}x$. The purpose of GE is to map each vertex of graph into a low-dimensional space that preserves the similarity between the vertex pairs. Then an optimal low-dimensional embedding is given by the graph preserving criterion as:

(4) $$\begin{array}{ll} V^{*} & =\underset{V^{T}XL_{p}X^{T}V=I}{\arg\;\min}\underset{i\neq j}{\sum }\|V^{T}x_{i}-V^{T}x_{j}{\|}^{2}W_{ij}\\ & =\underset{V^{T}XL_{p}X^{T}V=I}{\arg\;\min}\mathrm{tr}\left(V^{T}XLX^{T}V\right) \end{array}$$

where L=D−W is the Laplacian matrix. D is a diagonal matrix with $D_{ii}=\sum_{j=1}^{N}W_{ij}$. The weight $W_{ij}$ is used to measure the similarity of the edge connecting vertices. $L^{P}$ is the Laplacian matrix of the penalty graph $G^{P}$ or a simple scale normalization constraint. The linearization extension of graph embedding is computationally efficient for both projection learning and final classification. The construction of intrinsic graph and penalty graph becomes the crux of most dimensionality reduction methods. Besides, the intrinsic and penalty graphs could be, as our method shows, the intra-class and inter-class graphs.

## 3. Proposed Method

In Section 3.1, we will detail the two steps of BLSE, i.e., BLSR and BLSGE. The optimization processes for BLSR and BLSGE will be given in Section 3.2. Section 3.3 describes the classification process.

### 3.1. Block-Diagonal Constrained Low-Rank and Sparse Based Embedding (BLSE)

#### 3.1.1. Block-Diagonal Constrained Low–Rank and Sparse Representation (BLSR)

**Figure 3:**
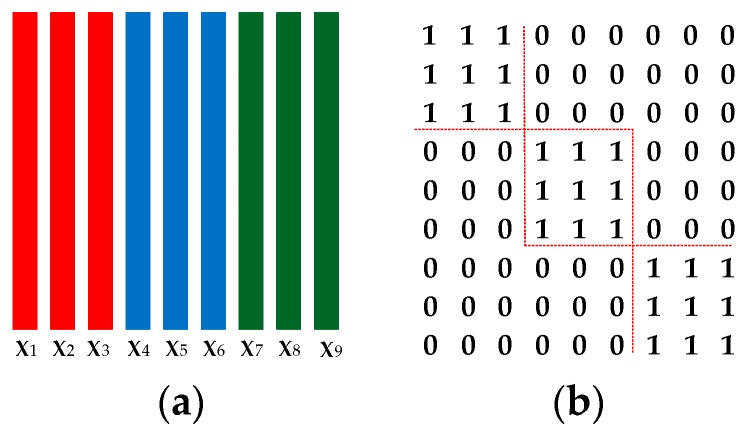
An illustration for the definition of matrix M. (**a**) 9 training samples from 3 classes with 3 samples per class; (**b**) The defined mask matrix M. If $l\left(x_{i}\right)=l\left(x_{j}\right)$, $M_{i,j}=1$, $M_{j,i}=1$, otherwise $M_{i,j}=0$, $M_{j,i}=0$. The diagonal-block elements of M are all ones, and the rest are zeros.

To reveal the intra-class and inter-class adjacent relationships and discover the local and global structures in data, a self-expressive model, i.e., BLSR is firstly developed. The label information of data is harnessed by introducing a block-diagonal constraint to purse a block-diagonal solution. Specifically, the BLSR model is formulated as:

(5) $$\underset{Z,E}{\min}\;{\left\|Z\right\|}_{*}+\frac{\alpha }{2}\|Z-Z⊙M{\|}_{F}^{2}+\beta {\left\|Z\right\|}_{1}+\gamma {\left\|E\right\|}_{1}\;s.t.\;X=XZ+E,\mathrm{diag}\left(Z\right)=0$$

where α, β and γ are the trade-off parameters for each component, and ${\left\|\cdot \right\|}_{F}$ denotes the Frobenius norm of a matrix. In (5), we try to discover the block-diagonal structure of the resolution via the block-diagonal regularization $\|Z-Z⊙M{\|}_{F}^{2}$, where ⊙ is the Hadamard product operator of matrices and M is a predefined mask matrix with an ideal block-diagonal structure. Figure 3 shows an example for the definition of M. Z⊙M is used to extract the intra-class representation coefficients for each sample. By minimizing $\|Z-Z⊙M{\|}_{F}^{2}$, representation coefficient of each sample corresponding to the inter-class samples is promoted to be small, but not necessarily be zero. Each sample is encouraged to be represented by the intra-class samples. The obtained block-diagonal representation matrix Z has good identification capability highlighting both the intra-class similarities and inter-class differences.

#### 3.1.2. Block-Diagonal Constrained Low–Rank and Sparse Graph Embedding (BLSGE)

The representation matrix Z of BLSR is then employed as affinity weights matrix to construct intra-class graph $G^{\mathrm{intra}}=\left\{X,W^{\mathrm{intra}}\right\}$ and inter-class graph $G^{\mathrm{inter}}=\left\{X,W^{\mathrm{inter}}\right\}$. First, we define the affinity matrix as $W=\left(\left|Z\right|+\left|Z^{T}\right|\right)/2$, then the connecting weights for intra-class and inter-class graphs are respectively defined as:

(6) $$W_{ij}^{\mathrm{intra}}=\{\begin{array}{c} W_{ij},\;\mathrm{if}\;W_{ij}\neq 0\;\mathrm{and}\;l\left(x_{i}\right)=l\left(x_{j}\right)\\ 0,\;\mathrm{otherwise} \end{array}$$

(7) $$W_{ij}^{\mathrm{inter}}=\{\begin{array}{c} W_{ij},\;\mathrm{if}\;W_{ij}\neq 0\;\mathrm{and}\;l\left(x_{i}\right)\neq l\left(x_{j}\right)\\ 0,\;\mathrm{otherwise} \end{array}$$

Whether two arbitrary points in the graphs are connected or not and the connection weight are adaptively determined by our BLSR model. It is desired that samples from the same class in feature space should be as close as possible, and those from different classes should be as far as possible. With projection matrix V, the optimization objective functions are defined as:

(8) $$\{\begin{array}{c} \min_{V}\frac{1}{2}\underset{i,j}{\sum }\|V^{T}x_{i}-V^{T}x_{j}{\|}^{2}W_{ij}^{\mathrm{intra}}\\ \max_{V}\frac{1}{2}\underset{i,j}{\sum }\|V^{T}x_{i}-V^{T}x_{j}{\|}^{2}W_{ij}^{\mathrm{inter}} \end{array}$$

For ease of classification, a big $W_{ij}^{\mathrm{intra}}$ is required in (8) to make the projected samples from the same class close to each other, and a small $W_{ij}^{\mathrm{inter}}$ is needed in (8) to make the projected samples from different classes far away from each other. The requirements can be satisfied by the representation strategy in BLSR. With some mathematical operations, we have:

(9) $$\{\begin{array}{c} \min_{V}\;\mathrm{tr}\left(V^{T}XL^{\mathrm{intra}}X^{T}V\right)\\ \max_{V\;}\mathrm{tr}\left(V^{T}XL^{\mathrm{inter}}X^{T}V\right) \end{array}$$

Then, the objective function of BLSGE can be formulated as:

(10) $$\min_{V}\;\frac{\mathrm{tr}\left(V^{T}XL^{\mathrm{intra}}X^{T}V\right)}{\mathrm{tr}\left(V^{T}XL^{\mathrm{inter}}X^{T}V\right)}$$

### 3.2. Optimizations for BLSR and BLSGE

#### 3.2.1. Optimization for BLSR

We convert problem (5) into the following equivalent problem by introducing auxiliary variables J and L:

(11) $$\underset{Z,E,J,L}{\min}\;{\left\|J\right\|}_{*}+\frac{\alpha }{2}\|Z-Z⊙M{\|}_{F}^{2}+\beta {\left\|L\right\|}_{1}+\lambda {\left\|E\right\|}_{1}\;s.t.\;X=XZ+E,Z=J,Z=L$$

We then have the corresponding Augmented Lagrange Multipliers (ALM) [41] function:

(12) $$\begin{array}{ll} \Xi & =\underset{Z,E,J,L}{\min}\;{\left\|J\right\|}_{*}+\frac{\alpha }{2}\|Z-Z⊙M{\|}_{F}^{2}+\beta {\left\|L\right\|}_{1}+\lambda {\left\|E\right\|}_{1}+\langle Y_{1},X-XZ-E\rangle \\ & +\langle Y_{2},Z-J\rangle +\langle Y_{3},Z-L\rangle +\frac{\mu }{2}\left(\|X-XZ-E{\|}_{F}^{2}+\|Z-J{\|}_{F}^{2}+\|Z-L{\|}_{F}^{2}\right) \end{array}$$

where $Y_{1}\in {ℜ}^{D\times n}$, $Y_{2}\in {ℜ}^{n\times n}$ and $Y_{3}\in {ℜ}^{n\times n}$ are Lagrange multipliers and μ>0 is a penalty parameter. The problem can be solved iteratively by updating each variable with others fixed. The steps to solve the problem in (*k* + 1)-th iteration are as follows:

*Step 1* (Update Z): Z can be updated by solving the following optimization problem (13):

(13) $$\begin{array}{ll} Z^{k+1}= & \underset{Z}{\mathrm{argmin}}\;\frac{\alpha }{2}\|Z-Z⊙M{\|}_{F}^{2}+\langle Y_{1}^{k},X-XZ-E^{k}\rangle +\langle Y_{2}^{k},Z-J^{k}\rangle \\ & +\langle Y_{3}^{k},Z-L^{k}\rangle +\frac{\mu^{k}}{2}\left(\|X-XZ-E^{k}{\|}_{F}^{2}+\|Z-J^{k}{\|}_{F}^{2}+\|Z-L^{k}{\|}_{F}^{2}\right) \end{array}$$

Since the sub-problem for Z involves Hadamard product operator, which makes the problem hard to optimize. Alternatively, the Z in Z⊙M can be obtained from former iteration. Thus, an iterative algorithm can be formed to solve the sub-problem of Z, we have:

$$\begin{array}{cc} Z^{k+1} & =\underset{Z}{\mathrm{argmin}}\;\frac{\alpha }{2}\|Z-Z^{k}⊙M{\|}_{F}^{2}+\langle Y_{1}^{k},X-XZ-E^{k}\rangle +\langle Y_{2}^{k},Z-J^{k}\rangle +\langle Y_{3}^{k},Z-L^{k}\rangle \\ & \;+\frac{\mu^{k}}{2}\left(\|X-XZ-E^{k}{\|}_{F}^{2}+\|Z-J^{k}{\|}_{F}^{2}+\|Z-L^{k}{\|}_{F}^{2}\right) \end{array}$$

(14) $$\begin{array}{c} Z^{k+1}=\underset{Z}{\mathrm{argmin}}\;\frac{\alpha }{2}\|Z-Z^{k}⊙M{\|}_{F}^{2}\\ +\frac{\mu^{k}}{2}\left(\|X-XZ-E^{k}+\frac{Y_{1}^{k}}{\mu^{k}}{\|}_{F}^{2}+\|Z-J^{k}+\frac{Y_{2}^{k}}{\mu^{k}}{\|}_{F}^{2}+\|Z-L^{k}+\frac{Y_{3}^{k}}{\mu^{k}}{\|}_{F}^{2}\right)\\ ={\left(\left(\alpha /\mu^{k}+2\right)I+X^{T}X\right)}^{-1}(X^{T}\left(X-E^{k}\right)+J^{k}+L^{k}\\ +\left(\alpha \left(Z^{k}⊙M\right)+X^{T}Y_{1}^{k}-Y_{2}^{k}-Y_{3}^{k}\right)/\mu^{k}) \end{array}$$

*Step 2* (Update E): E can be updated by solving following optimization problem (15):

(15) $$E^{k+1}=\underset{E}{\mathrm{argmin}}\;\lambda {\left\|E\right\|}_{1}+\langle Y_{1}^{k},X-XZ^{k+1}-E\rangle +\frac{\mu^{k}}{2}\|X-XZ^{k+1}-E{\|}_{F}^{2}\;=\underset{E}{\mathrm{argmin}}\;\lambda {\left\|E\right\|}_{1}+\frac{\mu^{k}}{2}\|X-XZ^{k+1}-E+\frac{Y_{1}^{k}}{\mu^{k}}{\|}_{F}^{2}=S_{\lambda /\mu^{k}}\left(X-XZ^{k+1}+\frac{Y_{1}^{k}}{\mu^{k}}\right)$$

*Step 3* (Update J): J can be updated by solving the following optimization problem:

(16) $$J^{k+1}=\underset{J}{\mathrm{argmin}}\;{\left\|J\right\|}_{*}+\frac{\mu^{k}}{2}\|Z^{k+1}-J+\frac{Y_{2}^{k}}{\mu^{k}}{\|}_{F}^{2}=US_{1/\mu^{k}}\left[\Sigma \right]V^{T}$$

where $\left(U,\Sigma ,V^{T}\right)=\mathrm{SVD}\left(Z^{k+1}+\frac{Y_{2}^{k}}{\mu^{k}}\right)$, $S_{\varepsilon }\left[\cdot \right]$ is the soft-thresholding (shrinkage) operator given by:

(17) $$S_{\varepsilon }\left[x\right]=\{\begin{array}{c} x-\varepsilon ,\;if\;x>\varepsilon \\ x+\varepsilon ,if\;x<-\varepsilon \\ 0,\;\mathrm{otherwise} \end{array}$$

*Step 4* (Update L): L can be updated by solving following optimization problem (18):

(18) $$L^{k+1}=\underset{L}{\mathrm{argmin}}\;\beta {\left\|L\right\|}_{1}+\frac{\mu^{k}}{2}\|Z^{k+1}-L+\frac{Y_{3}^{k}}{\mu^{k}}{\|}_{F}^{2}=S_{\beta /\mu^{k}}\left(Z^{k+1}+\frac{Y_{3}^{k}}{\mu^{k}}\right)$$

*Step 5*: Update the multipliers and μ:

(19) $$\{\begin{array}{c} Y_{1}^{k+1}=Y_{1}^{k}+\mu^{k}\left(X-XZ^{k+1}-E^{k+1}\right)\\ Y_{2}^{k+1}=Y_{2}^{k}+\mu^{k}\left(Z^{k+1}-J^{k+1}\right)\\ \;Y_{3}^{k+1}=Y_{3}^{k}+\mu^{k}\left(Z^{k+1}-L^{k+1}\right)\\ \mu^{k+1}=\min\left({\rho \mu }^{k},\mu_{max}\right) \end{array}$$

**Algorithm 1.** Solving BLSR by Inexact ALM**Input**: Training data X. Parameters α, β and λ.**Initialization**: $Z^{0}=J^{0}=E^{0}=0$, $Y_{1}^{0}=Y_{2}^{0}=Y_{3}^{0}=0$,  $\mu^{0}=10^{-5}$, $\mu_{max}=10^{8}$, $\varepsilon =10^{-6}$, ρ=1.1.1: **While** not converged **do**2: Fix other variables and optimize $Z^{k+1}$ via (14).3: Fix other variables and optimize $E^{k+1}$ via (15).4: Fix other variables and optimize $J^{k+1}$ via (16).5: Fix other variables and optimize $L^{k+1}$ via (18).6: Update the multipliers and μ via (19).7: Check the convergence conditions:  $\|X-XZ^{k+1}-E^{k+1}{\|}_{\infty }<\varepsilon$, $\|Z^{k+1}-L^{k+1}{\|}_{\infty }<\varepsilon$  $\|Z^{k+1}-J^{k+1}{\|}_{\infty }<\varepsilon$8: **End while****Output**: Z

Generally, we outline the optimization process of BLSR in Algorithm 1. The major computational burden of BLSR is solving (14) and (16) because they involve matrix inversion and singular value decomposition (SVD). The overall computational complexity of BLSR is $O\left(\tau \left(n^{2}D+n^{3}\right)\right)$, where τ is the iteration number, and n is the number of training samples.

#### 3.2.2. Optimization for BLSGE

The trace-ratio problem in the form of (10) does not have a closed-form solution [27]. Consequently, such problem can be approximately solved as a determinant-ratio problem, so we turn to solve the following problem (20):

(20) $$\min_{V}\frac{\left|V^{T}XL^{\mathrm{intra}}X^{T}V\right|}{\left|V^{T}XL^{\mathrm{inter}}X^{T}V\right|}$$

With the method of Lagrangian multiplier, the solution of problem (20) is transformed to solve a generalized eigenvalues problem as follows:

(21) $$XL^{\mathrm{intra}}X^{T}V=\lambda \;XL^{\mathrm{inter}}X^{T}V$$

Then we obtain the eigenvectors corresponding to the d minimum eigenvalues, and the projection matrix can be got as $V=\left[v_{1},v_{2},\ldots ,v_{d}\right]$. The detailed process of BLSGE is given in Algorithm 2. The complete procedure of BLSE is outlined in Algorithm 3.
**Algorithm 2. BLSGE****Input**: Affinity weights matrix
Z, reduced dimension d.1: Compute the weights of inter-class graph (6) and intra-class graph (7) through affinity matrix Z. 2: Solve the generalized eigenvalue problem (21), and get the eigenvectors corresponding to the d minimum eigenvalues.**Output**: Projection matrix V.
**Algorithm 3. BLSE****Input**: labeled training data
$X\in {ℜ}^{D\;\times \;n}$. Reduced dimension d.    Tradeoff parameters α, β and γ.1: Run **Algorithm 1** to get the affinity weights matrix Z of X.2: Run **Algorithm 2** to obtain the optimal projection matrix V.**Output**: Projection matrix V.

### 3.3. Classification

For classification, we directly use the calculated projection V to obtain the transformation results of the training and testing data. One can apply existing classifier such as 1-Nearest Neighbor (NN) to classify the projected results of testing data.

## 4. Experimental Results

### 4.1. Analysis of BLSE

**Figure 4:**
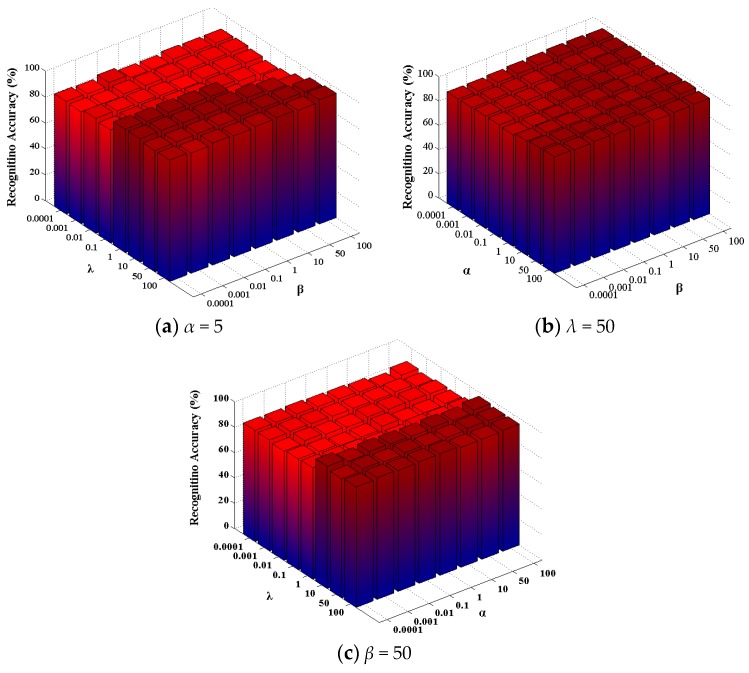
Sensitivity analysis of *α*, *β* and *λ* on ORL dataset. (**a**) Tuning *β* and *λ* with *α* fixed; (**b**) tuning *α* and *β* with *λ* fixed; (**c**) tuning *α* and *λ* with *β* fixed.

The representation results of BLSR have a great influence on the graph construction and the performance of BLSE. There are three parameters in BLSR, i.e., regularization parameters *α*, *β* and *λ*. *λ* and *β* controls the sparsity noise term E and representation Z. α is used to regularize the representation to be block-diagonal. We conduct experiments to study the sensitivity of the proposed BLSE over a wide range of these parameters. ORL data [42] are divided into training and testing samples for tuning these parameters, and the reduced dimension is 50. The experimental results obtained are used to find an effective range of parameters to ensure a reliable performance. The results are reported in Figure 4. From the results, one can observe that the performance of BLSE is not especially sensitive to *λ* and is robust in a quiet large range for *α* and *β*.

**Figure 5:**
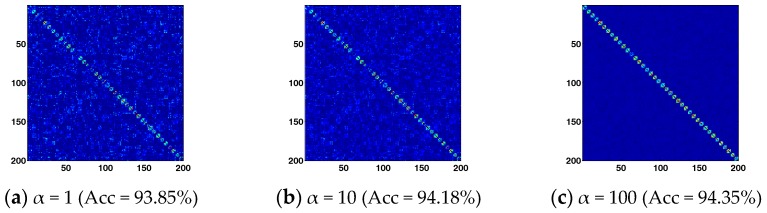
Visualization of the graph weights and corresponding recognition accuracy obtained by BLSE. From left to right, *α* is 1, 10 and 100, respectively.

Figure 5 further shows the graph weights matrix obtained by BLSR and the corresponding recognition accuracy obtained by BLSE with α = 1, 10, and 100 respectively. A larger penalty will be imposed on the block-diagonal regularization term as α increases. The obtained graph weights matrix tends to show a better block-diagonal structure. Benefitting from the block-diagonal graph weights matrix, BLSE achieves higher recognition accuracy. The results demonstrate that the proposed method can enhance the block-diagonal structure of graph weights matrix, which helps achieve better recognition performance. However, an extremely large α will regularize the inter-class representation in Z to be zero, which might not be able to reveal the inter-class adjacent relationship among samples well. To achieve reliable and stable performance, a suggested parameter settings are 100 > *α* >1, 50 > *β* > 0.01, 50 > *λ* >1.

### 4.2. 2-D Visualization Experiment on CMU PIE Dataset

**Figure 6:**
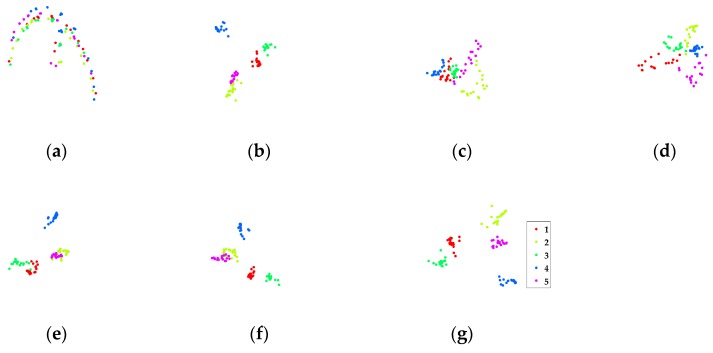
Two-dimensional five-class CMU PIE data projected by different DR methods. (**a**) PCA; (**b**) LDA; (**c**) MMC; (**d**) SGDA; (**e**) LGDA; (**f**) SLGDA; (**g**) BLSE.

In this part, a partial CMU PIE face database [43] (120 images of five persons) is used to intuitively show the discriminate ability of different methods using t-SNE [44]. In the experiment, seven images per person are randomly selected for training, and the remaining about 17 images for testing. Figure 6a–g visualize the testing data distributions along the first two dimensions acquired by different methods. From Figure 6, we may draw several conclusions. First, as classical supervised DR methods, LDA and MMC can yield superior performance to that of unsupervised PCA. Second, SGDA [27] only exploits the local neighborhood structure via sparse representation, and it does not perform well, as shown in Figure 6d. Some parts of class 1, 2, 3 and 4 mix together. By introducing global low-rank regularization, LGDA [28] shows better separation ability. Nevertheless, there are overlaps between class 2 and class 5, class 1 and class 3. With both sparse and low-rank constraints, SLGDA [28] performs better than SGDA and LGDA. However, class 2 and class 5 still have significant overlaps as shown in Figure 6f. Contrastively, the proposed BLSE successfully separates all the classes with clear boundaries between them, which can be explained by the simultaneously imposed local sparse, global low-rank and the discriminative block-diagonal structure constraint. The experiment shows that BLSE has the capacity to separate complex face data distribution.

### 4.3. Experimental Results on Image Datasets

**Figure 7:**
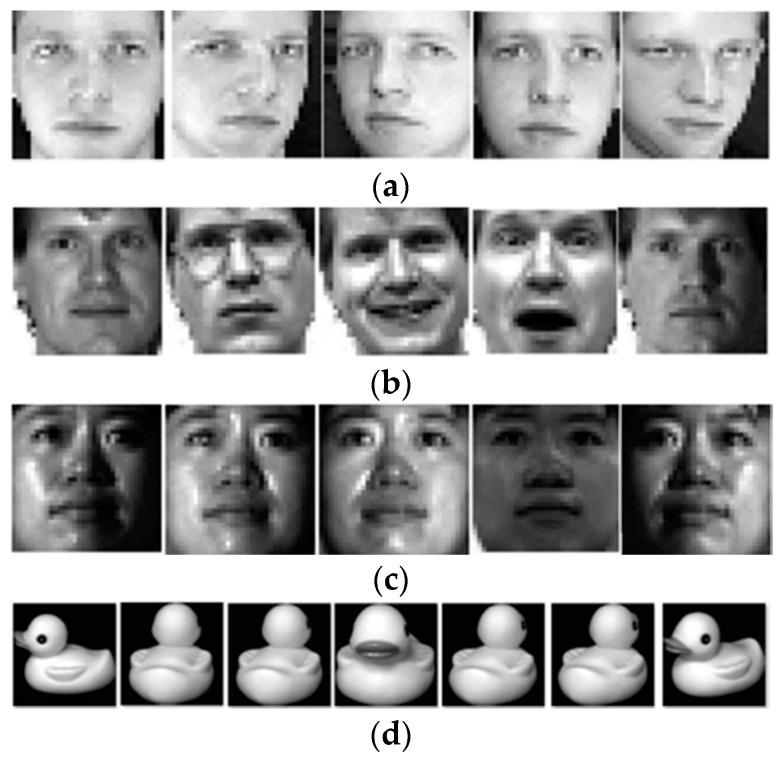
The sample images of public datasets used in our experiment. (**a**) The ORL dataset; (**b**) The Yale database; (**c**) The CMU PIE dataset; (**d**) The COIL20 dataset.

We conducted extensive experiments to evaluate the performance of the proposed method on widely used face and object databases (ORL [42], Yale [45], CMU PIE [43], and COIL 20 [46]). Figure 7 visually demonstrates characteristics of each database. Our approach is compared with several state-of-the-art subspace learning approaches including PCA, LDA, MMC [6], SGDA [27], LGDA [28], and SLGDA [28]. To make the comparison fair, for all the evaluated algorithms we first apply PCA as preprocessing step by retaining 99% energy. A nearest neighbor classifier is employed in the projected feature space for all the methods.

**Figure 8:**
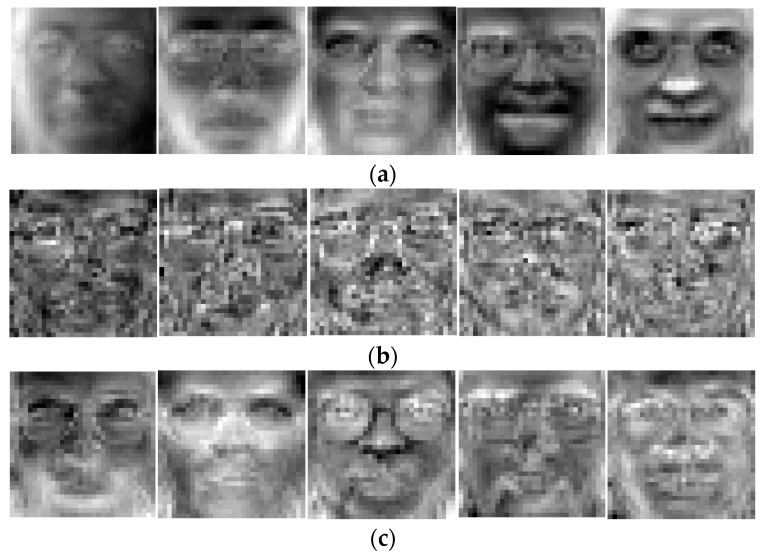
The first five basis vectors calculated by (**a**) PCA (Eigenfaces); (**b**) LDA (Fisherfaces); (**c**) BLSE (BLSEFaces) on ORL dataset.

The ORL face database consists of a total of 400 face images from 40 individuals with 10 images per person. The images were taken at different times, lighting variation, facial expressions (open/closed eyes, smiling/not smiling) and facial details (glassed/no glassed) against a dark homogeneous background. In the experiments, each image in ORL database is manually cropped and resized to 32 × 32. Using the five samples per person from ORL database as training set, we present the first five basis vectors of Eigenfaces, Fisherfaces, and our BLSEFaces in Figure 8.

A random subset with *t* (=3, 4, 5, 6) images of each individual is selected for training and the rest for testing. For each *t*, we run the programs 10 times and calculate the recognition rates as well as the standard deviations with different reduced dimensions.

The Yale face database contains 165 gray scale images of 15 individuals, each individual has 11 images. The images demonstrate variations in lighting condition, facial expression (normal, happy, sad, sleepy, surprised, and wink). In our experiments, each image in Yale database was manually cropped and resized to 32 × 32. A random subset with *t* (=4, 5, 6, 7) images each individual is selected for learning the embedding and the rest for testing. For each giving *t*, we run each program 10 times to randomly choose the training set and report the average recognition rates as well as the standard deviations with different reduced dimensions.

The CMU PIE dataset contains over 40,000 face images of 68 individuals. Images of each individual were acquired across 13 different poses under 43 different illumination conditions, and with four different expressions. Here we use a near frontal pose subset, namely C07, for experiments, which contains 1629 images of 68 individuals. Each individual has about 24 images. All images are manually cropped and resized to 32 × 32 pixel. A random subset with *t* (=4, 5, 6, 7) images for each individual is selected for learning the embedding and the rest for testing. For each giving *t*, we perform 10 times to randomly choose the training set and report the average recognition rates as well as the standard deviations under different dimensions.

The COIL20 image dataset contains 1440 gray scale images of 20 objects with 72 images per subject. The images of each object were taken 50 apart as the object was rotated on a turntable. Each image is of size 32 × 32. Following the experimental setting in [47], we selected the first 36 images per subject for training and the remaining images for testing in this experiment.

**Figure 9:**
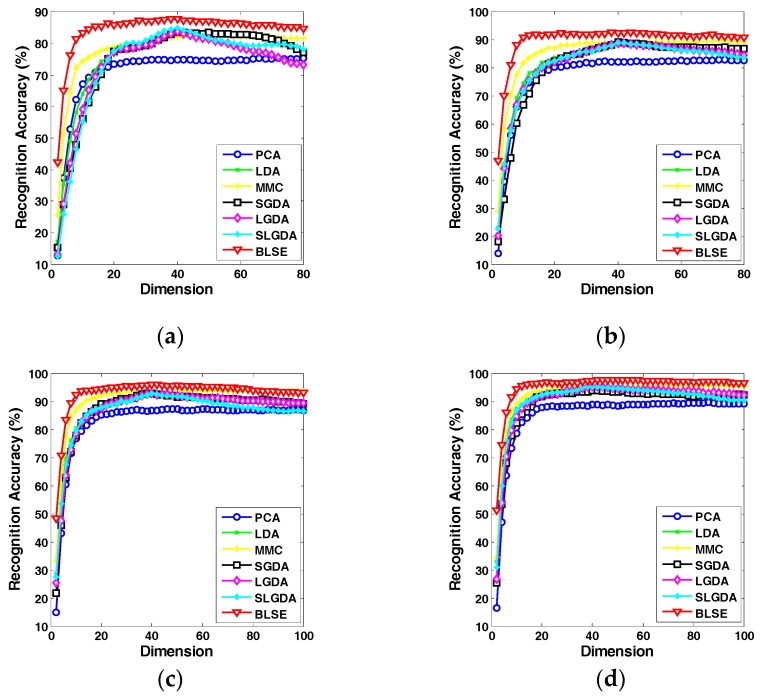
Recognition rate (%) versus dimension of different methods on the ORL database. (**a**) Three training samples per person; (**b**) Four training samples per person; (**c**) Five training samples per person; (**d**) Six training samples per person.

**Figure 10:**
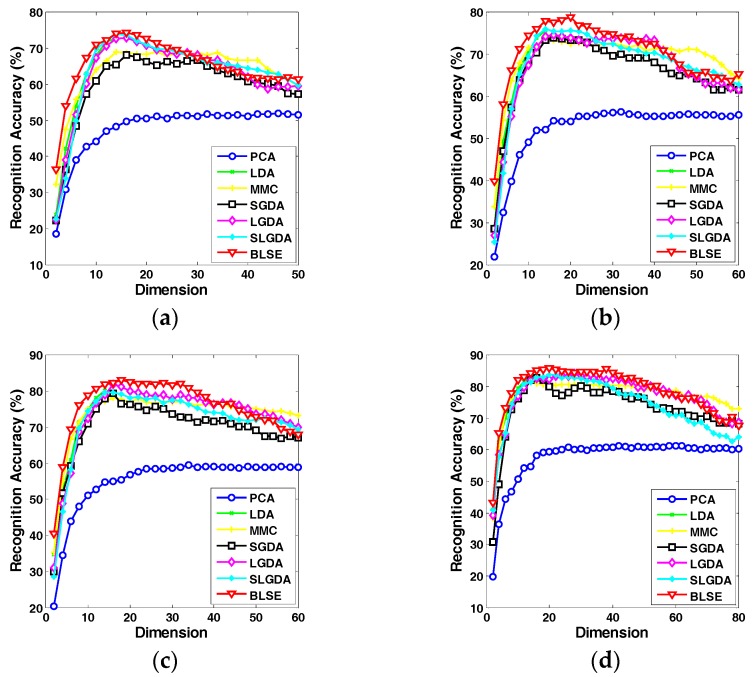
Recognition rate (%) versus dimension of different methods on the Yale dataset. (**a**) Four training samples per person; (**b**) Five training samples per person; (**c**) Six training samples per person; (**d**) Seven training samples per person.

**Figure 11:**
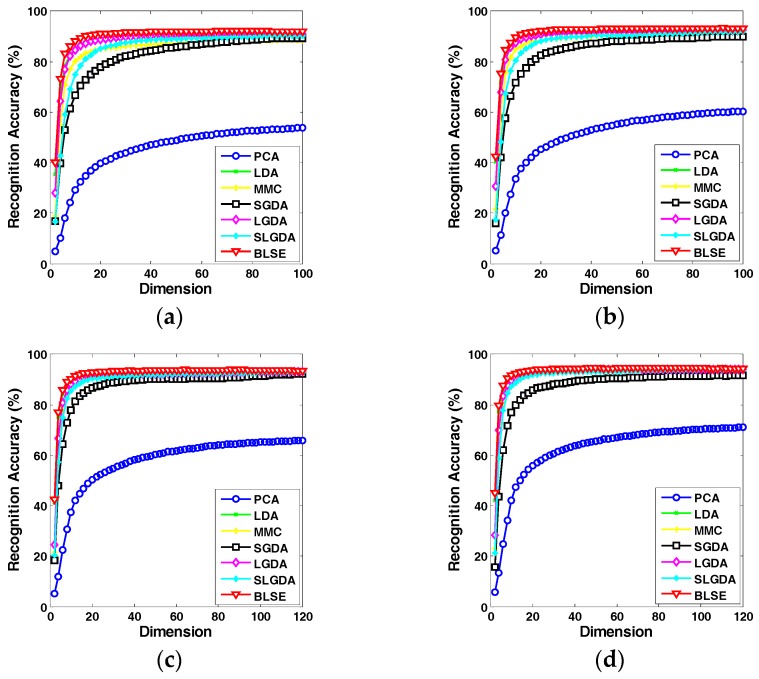
Recognition rate (%) versus dimension of different methods on the CMU PIE dataset. (**a**) Four training samples per person; (**b**) Five training samples per person; (**c**) Six training samples per person; (**d**) Seven training samples per person.

**Figure 12:**
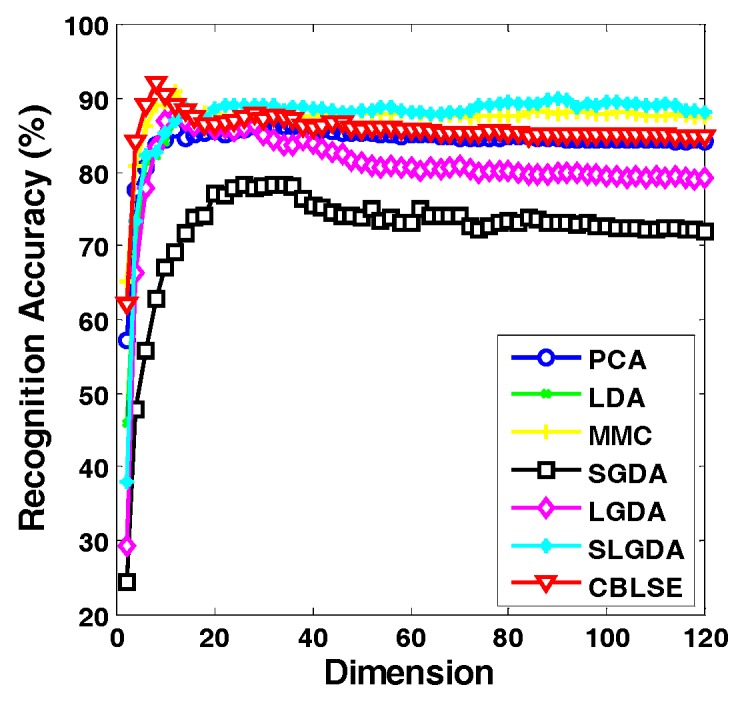
Recognition rate (%) versus dimension of different methods on the COIL20 database.

Figure 9, Figure 10, Figure 11 and Figure 12 plot the curves of average recognition accuracy versus different dimensions on ORL, Yale, CMU PIE and COIL 20 databases, respectively. Moreover, the details of experiments results, namely maximal recognition rates together with the standard deviation and dimension of different algorithms are summarized in Table 1.

## 5. Discussion and Conclusions

Based on the experimental results on face and object image datasets, one can conclude that with the increase of dimensions and the number of training samples per class, all the methods tend to achieve better performance. PCA is simple to calculate, and performs well in some cases, but its unsupervised nature restricts its performance. By introducing supervised information with different discrimination criteria, LDA and MMC can achieve better performance. SGDA, LGDA and SLGDA can adaptively select neighbors for graph construction, and find the representation of each sample using the labeled samples in the same class to purse block-diagonal structure representations. However, this process may result in large representation error due to the limited samples per class, which might not be able to reveal the intra-class adjacent relationship well. Besides, SGDA, LGDA and SLGDA disconnect inter-class samples in graph construction. The operation cannot capture the inter-class adjacent relationship well. As a result, SGDA, LGDA and SLGDA do not perform well, as the experimental results show. Comparably, the proposed BLSE model can achieve better performance. The reason is twofold. Firstly, the developed BLSR method can capture both local neighborhood relations and global structures latent in data with low-rank and sparse constraints. Different from SGDA, LGDA and SLGDA, all samples are employed in BLSR when finding the representation of each sample. The introduction of block-diagonal regularization can capture the intra-class and inter-class adjacent relationships hidden in data, and enhance the identification capability of BLSR. Secondly, benefit from BLSR and GE framework, the discriminative capacity of low dimensional subspace learned by BLSGE is further boosted by simultaneously minimizing the intra-class scatter and maximizing the inter-class scatter.

To conclude, we have proposed a novel block-diagonal constrained low-rank and sparse based embedding (BLSE) model for the dimensionality reduction and classification of image data. Two procedures of BLSE, namely, block-diagonal constrained low-rank and sparse representation (BLSR) and block-diagonal constrained low-rank and sparse graph embedding (BLSGE), are detailed. BLSR takes the advantages of local discriminative capacity of SR and the global low-rank property of LRR. Meanwhile, a novel block-diagonal regularization term is introduced to fully harness the label information and purse a block-diagonal representation. The affinity weights matrix obtained by BLSR can well reveal the intra-class similarities and inter-class differences of data. With the intra-class and inter-class graphs derived from BLSR, BLSGE finds a low-dimensional subspace with enhanced intra-class compactness and inter-class separation. Experimental results on public face and object datasets are performed, and validate the effectiveness of BLSE model.

## Figures and Tables

**Table 1:** Recognition rates (%) and the corresponding standard deviations and dimensions (in parenthesis) on ORL, Yale, CMU PIE and COIL20 databases.

Dataset	*t*	Compared Methods						Ours
		PCA	LDA	MMC	SGDA	LGDA	SLGDA	BLSE
ORL	3	75.32 ± 2.58 (80)	83.63 ± 2.38 (38)	82.39 ± 2.73 (56)	83.61 ± 2.72 (40)	83.79 ± 2.15 (40)	84.96 ± 1.78 (40)	**87.68 ± 2.36 (38)**
	4	82.92 ± 1.79 (72)	88.71 ± 2.26 (38)	89.58 ± 2.44 (72)	89.42 ± 2.08 (40)	88.83 ± 2.36 (40)	89.08 ± 2.63 (40)	**92.63 ± 2.28 (40)**
	5	87.45 ± 1.70 (48)	93.15 ± 1.13 (38)	93.95 ± 1.55 (64)	93.10 ± 1.94 (40)	93.00 ± 0.91 (40)	92.95 ± 1.41 (40)	**96.05 ± 0.96 (42)**
	6	89.75 ± 1.65 (86)	95.75 ± 1.31 (38)	95.81 ± 1.22 (70)	94.06 ± 1.48 (40)	95.50 ± 1.09 (40)	95.44 ± 1.22 (40)	**97.88 ± 0.67 (44)**
Yale	4	52.00 ± 2.34 (46)	72.76 ± 2.30 (14)	69.05 ± 3.51 (14)	68.29 ± 3.81 (16)	72.86 ± 2.16 (16)	73.90 ± 2.92 (14)	**74.38 ± 2.48 (16)**
	5	56.33 ± 5.57 (32)	75.67 ± 2.31 (14)	73.44 ± 4.52 (14)	74.00 ± 3.52 (16)	74.56 ± 3.33 (16)	76.11 ± 2.83 (14)	**78.89 ± 3.10 (20)**
	6	59.60 ± 5.76 (34)	80.27 ± 3.81 (14)	78.13 ± 5.56 (16)	79.47 ± 3.51 (16)	81.87 ± 3.51 (16)	79.60 ± 5.34 (14)	**83.20 ± 4.71 (18)**
	7	61.33 ± 5.02 (42)	83.00 ± 2.70 (14)	81.67 ± 4.30 (14)	82.83 ± 3.34 (16)	84.17 ± 4.10 (28)	83.83 ± 4.45 (22)	**85.83 ± 3.17 (20)**
CMU PIE	4	53.72 ± 1.28 (100)	91.14 ± 1.25 (64)	88.61 ± 1.50 (96)	89.44 ± 1.35 (100)	91.33 ± 0.81 (86)	90.40 ± 1.03 (94)	**92.28 ± 1.07 (80)**
	5	60.31 ± 1.78 (100)	92.60 ± 0.83 (66)	91.27 ± 0.85 (98)	89.95 ± 1.84 (98)	92.60 ± 0.83 (66)	91.82 ± 0.99 (100)	**93.26 ± 1.03 (92)**
	6	65.88 ± 1.92 (120)	93.56 ± 0.88 (66)	93.17 ± 1.03 (106)	92.15 ± 0.95 (112)	93.24 ± 1.04 (104)	92.84 ± 0.92 (118)	**93.93 ± 1.01 (92)**
	7	71.12 ± 1.61 (120)	94.34 ± 0.83 (66)	94.09 ± 0.66 (110)	93.54 ± 0.58 (120)	94.09 ± 0.72 (104)	93.88 ± 0.59 (102)	**94.64 ± 0.59 (70)**
COIL20	36	86.39 (28)	88.75 (14)	90.97 (12)	78.33 (26)	87.78 (12)	90.00 (90)	**92.22 (8)**
